# Supplementary material for: Are carbon emissions trading and green financial instruments synergistic? -Comprehensive quantitative research based on content analysis
Source: PLoS One. 2024 Mar 7;19(3):e0298601. doi: 10.1371/journal.pone.0298601 (PMC10919601; doi:10.1371/journal.pone.0298601)
Supplement: S1 Data — (ZIP) [file pone.0298601.s001.zip › Data/地级市Z_F工作报告.zip!/║┌┴·╜¡/╞δ╞δ╣■╢√╩╨/└┤╫╘╣1⁄2╓┌║┼í╛┬φ┐╦╩2╛▌═°í┐_2019.docx]

各位代表：

　　现在，我代表市人民政府向大会报告工作，请予审议，并请市政协委员提出意见。

　　2018年，我们坚持以习近平新时代中国特色社会主义思想为指导，在市委正确领导下，坚决贯彻落实党的十九大和习近平总书记系列重要讲话以及对我省我市重要指示精神，贯彻新发展理念，突出高质量发展要求，加快构建以12个重点产业为支撑的现代产业体系，持续改善民生，经济社会稳步发展。预计全市地区生产总值同比增长6%以上；固定资产投资增长5%左右；公共财政预算收入和地方税收分别增长1.8%和5.4%（可比口径分别增长8.4%和11.5%）；城乡居民人均可支配收入增长6.5%以上；万元GDP综合能耗持续下降；完成主要污染物减排任务。

　　——农业市场化程度稳步提高。全面实施乡村振兴战略。农业获得大丰收，粮食总产增长10%以上。以市场为导向，调整种植结构，优化品种品质，“三大作物”稳定在3000万亩以上，蔬菜、食用菌、中草药等经济作物规模扩大。农业综合机械化率提高到97%，划定粮食生产功能区和重要农产品生产保护区2911万亩。新建“两牛一猪”规模养殖场18个。严密防控非洲猪瘟疫情。加快培育三产融合新型经营主体，新增农业示范公司30个、股份合作组织36个、一体化合作社与联合社63个；益海嘉里现代农业产业园等一批项目开工建设，飞鹤奶山羊养殖一期及乳品加工、阜丰二期玉米深加工等一批项目建成投产，市级以上农业产业化龙头企业达到233户。注重品牌建设和集中推广，强化线上线下一体化营销，国家生态原产地、绿色（有机）农产品认证达到521个，绿博会、好粮油中国行等展会成效明显，农产品电商上行销售额增长47.3%。

　　——工业经济提质增速。规上工业增加值增长10%以上，主营业务收入和利润分别增长20%和50%以上。装备制造、食品加工两大产业主营业务收入分别增长20%和35%以上，产业倍增计划加快实施。飞鹤乳业销售额突破百亿元，一重主营业务收入超过130亿元，建龙北钢产量突破百万吨，总规模超百亿元的中车齐车集团总部落户我市，紫金铜产业园等一批项目加快建设。“五化”示范带动效应显现，新增省级数字化示范企业3户。新增规上企业27户，央企地方配套率达到40%。促进降本增效，发放贷款周转金5.8亿元，减税降费5.6亿元，40户直供电企业节支1.6亿元，2支专项产业基金实质运营。我市“工业稳增长和转型升级”举措受到国务院通报表扬。

　　——服务业升级加快。社会消费品零售总额增长5%左右，外贸进出口总额增长1.2倍。限上企业新增46户，触网率达71%。引进浪潮、九次方等战略性互联网企业，电商注册企业突破千户，县区电商平台和园区实现全覆盖，新增2个国家级电子商务进农村示范县，上行销售额突破30亿元。进口肉类指定查验场等项目加快建设。旅游业发展加快，奥悦冰雪体育公园、鹤城游乐园等一批项目开工建设，万达嘉华酒店建成运营，碾子山全域旅游加快打造，成功举办鹤城国际冰球、全国山地自行车等系列赛事，带动百万人上冰雪游鹤城。线上线下一体化旅游服务体系建设加快，全年接待游客和旅游总收入分别增长11%和17%。推出《情系双龙湾》话剧等文艺作品80余部，商演800余场。齐齐哈尔农商行挂牌运营，各类资本市场挂牌企业117户。与广州对口合作签约项目58个。

　　——改革创新全面推进。深化简政放权，取消下放市级行政权力67项。一体化智慧政务体系初步建立，在全省率先启用政务大数据平台，“鹤城在线”APP上线运行；市级1256项政务服务事项“一窗办、一次办”比例达94.4%；在全省率先实现不动产登记“一窗受理、一网办结、一城同办”，最复杂事项办理不超过6个工作日；公共资源电子招投标平台通过国家三星认证。商事登记实现“三十五证合一”，新增市场主体2.5万户。社会信用体系建设全面启动，信用信息平台投入应用，推行失信联合惩戒机制。实施“三项制度”，完善重点领域“双随机一公开”执法机制。完成新一轮政府机构组建，事业单位分类改革有序推进。完成自然资源统一确权国家试点任务，基本完成农村土地确权颁证、宅基地确权试点、村集体资产清产核资和国有林场改革。驻齐央企“三供一业”移交有序推进，启动新一轮国企国资改革。全面取消公立医院药品加成并实施购销“两票制”。新增重点科创平台23个，新建科技企业孵化器8个、创新型产业联盟14个，新认定国家级高新企业7户、科技型中小企业86户。省科淘网齐齐哈尔子平台上线运行。科技成果转化应用386项。开发区改革创新全面启动；高新区在孵项目82个，成为国家高端人才引领型特色载体。

　　——生态环境治理明显加强。以前所未有的决心和力度，深入开展蓝天、碧水、净土三大保卫战，以中央环保督察“回头看”反馈和群众反映的突出问题为镜鉴，举一反三，全面彻底排查，实施台账式整改，生态环境治理强力推进。取缔燃煤小锅炉213台，淘汰高污染排放车辆2705台，新能源公交体系基本建成，全面实施秸秆禁烧，PM2.5平均浓度28微克/立方米，优良天数达到94.15%，空气质量明显提升。落实落靠河湖长制，建立“一江十河”管护机制和乌裕尔河、双阳河流域生态补偿机制，全面开展清河行动，排查整改河湖问题1886个，嫩江主城区段、劳动湖水生态修复工程加快推进，启动14个污水处理厂提标改造，浏园新水源建成投用，城区泄洪区污泥堆存、昊华电石渣堆场整改完成，红星垃圾场搬迁、昂昂溪纳污坑塘黑臭水体整治加快，城区污泥和餐厨垃圾处理项目建成运行，规模养殖场粪污处理设施配套率达90%以上，影响水环境的突出问题得到有效遏制。农业“三减”5%以上，耕地轮作试点300万亩，植树造林9.39万亩，“大棚房”整治取得阶段性成果，龙江、克山成为全省黑土地保护利用整建制试点县，土壤生态持续改善。自然保护区“绿盾行动”整改问题225个，拆违19.7万平方米，退耕还湿1万亩。查处生态违法案件121起。

　　——民生保障能力稳步提升。系统推进“七大民生工程”，民生投入占一般公共预算支出的88.6%。脱贫攻坚取得阶段性成果。富裕、甘南、克山、依安摘帽退出，龙江、克东、泰来达到退出验收条件。4.59万人实现脱贫，193个贫困村出列，贫困发生率降至0.96%。社会保障水平稳步提高。发放创业担保贷款2.4亿元、稳岗补贴3938万元，城镇新增就业10万人，城镇登记失业率控制在3.81%。城乡居民、企业职工养老金待遇每月人均分别提高28元和126元，市县城镇职工失业保险待遇每人每月分别提高280元和325元。城乡居民医保最高支付限额提高2万元，36种用量大、费用高的特殊用药纳入医保。发放各类救助资金9.6亿元，城乡低保标准提高3%，优抚待遇提高10%。市残疾人康复中心竣工。人居环境持续改善。棚改回迁安置1.24万户，改造农村危房1.29万户。中心城区改造老旧小区10个、低温楼104栋；5大新热源投入使用；升级改造市政道路23条，化工桥、文化北大桥开工建设，南外环、明月岛浮桥建成通车。城区新增绿地479亩，拆违3305处，城市亮化率明显提高。启动农村人居环境整治三年行动。新建农村道路1200余公里，解决2.6万贫困人口饮水安全问题，改灶7.6万户，改厕1万户，农村垃圾集中收集处理体系初步形成。社会事业稳步发展。建设幼儿园15所、标准化学校32所，“阳光分班”、校内课后服务受到学生家长普遍欢迎，义务教育基本均衡县建设全面完成，省部共建国家现代农村职教改革试点市通过验收。新增名医工作室10个，重点人群家庭医生签约率77.6%。新增养老床位2350张。市图书馆晋升一级馆，文化惠民演出260场，新增体育健身设施500余套。平安鹤城扎实推进。开展“扫黑除恶”专项斗争，刑事案件下降5.5%；不动产登记证补办等一批历史遗留和疑难问题得到化解；生产安全事故下降5%；新增城乡示范社区33个、国家级综合减灾示范社区4个。

　　——政府自身建设持续加强。深入学习贯彻习近平总书记系列重要讲话，认真开展解放思想大讨论。推进法治政府建设，建立学法制度，完善工作运行规则，提高依法行政能力。主动接受人大依法监督、政协民主监督及社会监督，办理人大代表建议179件、政协委员提案400件。深入推进作风整顿优化营商环境活动，集中整治窗口服务突出问题。深入落实党风廉政建设责任制，认真整改中央和省委巡视反馈问题；强化财政绩效管理和政府投资全过程审计监督，审查项目569个、审减资金2.7亿元，“三公经费”压减12%。地方政府债务总体可控。

　　双拥共建、民族宗教、外事侨务、防灾减灾、档案史志、妇女儿童、老龄、人防等工作都取得了新成绩。

　　各位代表！过去的一年，我们直面问题，勇于担当，拼搏攻坚，取得了来之不易的发展变化。这些发展变化，得益于习近平新时代中国特色社会主义思想的正确指引，得益于省委、省政府和市委的坚强领导，得益于市人大、市政协的监督支持，得益于社会各界和全市人民的团结奋进。在此，我代表市人民政府，向全市广大干部群众，向人大代表、政协委员，向各民主党派、工商联、无党派人士和人民团体，向驻齐部队、武警官兵、公安民警和中省直单位，向关心支持齐齐哈尔振兴发展的社会各界人士，表示衷心的感谢并致以崇高的敬意！

　　习近平总书记在深入推进东北振兴座谈会上指出东北地区存在“四大短板”，通过开展解放思想大讨论，对照查找我们的问题，具体表现为：在体制机制上，计划经济体制影响依然较深，政府习惯于用行政方式研究解决问题，不善于用市场机制优化营商环境、培育市场主体、提供公共服务，看病难看病贵、教育质量不高、物业服务差、供热等民生保障水平低、农村人居环境乱等问题尚未根本解决。市场主体特别是中小企业产权单一，企业制度不健全，“行政式”“家族式”管理粗放，竞争发展能力不强。在经济结构上，区域发展不协调，资源占比大的县域经济发展不充分。产业结构畸轻畸重，一产农头大食尾小、产量高效益低，二产行业和企业分化明显、主导产业配套率低，三产新业态新模式少，满足域内、辐射域外、引领消费能力弱。产品结构优化迟缓，多年一贯制、大众化、同质化的特征明显。在开放合作上，经济外向度不高，进出口带动作用弱，项目建设目的性、专业性、实效性、突破性差，缺乏“走出去、引进来”的底气；企业在资本、技术、产品上缺少优势互补、合作共赢的信心。产业联盟要素协同不够，企业融资难融资贵、用工荒依然突出。在思想观念上，诸多问题产生的总根源在于思想不解放。新发展理念不牢，全面协调可持续发展意识不强，公职人员缺乏将脱贫攻坚、生态环境整治的压力转化为推动振兴发展的动力；市场主体更多关注生产和经济利益，对生态环境保护重视不够。市场化意识不强，资源市场化配置不充分，公平公正公开的市场管理机制尚未形成；市场主体缺乏竞争精神和开拓力度。创新精神不足，公职人员缺乏破与立的勇气，不注重学习借鉴先进地区经验，习惯于墨守成规，安于现状；市场主体缺乏创新领先意识，产品、业态、模式习惯“跟风”。社会风气不够优化，缺乏依规行事的高度自觉，习惯于大事小事“托人情”“找关系”。对于这些问题，我们一定解放思想，精准施策，认真加以解决。

　　2019年是新中国成立70周年，是决胜全面建成小康社会的决定性之年，也是推进全面振兴全方位振兴的发力之年。习近平总书记再次深入东北三省特别是亲临我市考察，为我们指明了前进方向，提供了强大动力；国家新一轮东北振兴“1+N”、大力扶持民营经济等政策举措陆续出台，中央经济工作会议提出“六稳”“八字”方针，必将激发微观主体发展的更大活力；我市具有生态、土地、人力资源和产业基础比较优势，蕴藏着高质量发展的巨大潜力。总体看，我市正处于振兴发展的重要战略机遇期。只要我们保持定力，务实担当，就一定能够化危为机，变压力为动力，走出一条全面振兴全方位振兴高质量发展新路子。

　　政府工作的总体思路是：以习近平新时代中国特色社会主义思想为指导，全面贯彻党的十九大和十九届二中、三中全会精神，深入落实中央和省委经济工作会议、省委十二届四次全会和市委十三届四次全会精神，统筹推进“五位一体”总体布局和协调推进“四个全面”战略布局，坚持稳中求进工作总基调，坚持新发展理念，坚持以供给侧结构性改革为主线，坚持以全面振兴全方位振兴高质量发展为主题，继续打好“三大攻坚战”，努力建设“六个强市”，统筹推进稳增长、促改革、调结构、惠民生、防风险，落实“六稳”要求，深入解放思想，勇于担当作为，扬长避短、扬长克短、扬长补短，着力优化营商环境，着力促进产业振兴，着力保障生态安全，着力增强群众获得感幸福感安全感，着力提升现代治理能力，为鹤城全面振兴全方位振兴打牢坚实基础，以优异成绩庆祝新中国成立70周年。

　　主要预期目标是：全市地区生产总值增长6%以上；规上工业增加值增长10%以上；固定资产投资增长4%；公共财政预算收入增长2%以上；社会消费品零售总额增长6%；城乡居民人均可支配收入增长6.5%以上；城镇登记失业率控制在4%以内；万元GDP综合能耗下降3%；主要污染物减排任务全面完成。

　　做好政府工作，推动全面振兴全方位振兴高质量发展，要坚持以新发展理念为引领，坚定不移贯彻创新、协调、绿色、开放、共享发展理念，依靠科学发展实现局部振兴与全面全方位振兴相统一，经济社会与生态保护相统筹，城区与县域发展相协调，产业发展与科技创新、现代金融、人力资源相协同，军民发展相融合。要坚持以解放思想为先导，坚持问题导向，找准制约根源，以破与立的勇气深化改革，以改革倒逼思想大解放，在解放思想中攻坚克难、优化环境、突破发展。要坚持以市场化改革增强微观主体活力，更多采用改革创新的办法，更多运用市场化、法治化手段，把中央提出的“巩固、增强、提升、畅通”八字方针贯穿于经济发展各方面，把优化营商环境和创新驱动融入产业发展全过程，做好“三篇大文章”，着力激发微观主体活力，全面打造产业振兴发展新动能。要坚持以高质量项目推动产业振兴，把产业项目建设作为振兴发展的关键，围绕12个重点产业，下决心突破一批高质量产业项目，加快构建现代产业体系。要坚持以乡村振兴战略统领农业农村工作，把深入实施乡村振兴战略作为新时代“三农”工作的总抓手，大力推动产业振兴、人才振兴、文化振兴、生态振兴、组织振兴和农村人居环境改善。要坚持以人民为中心保障和改善民生，持之以恒为群众办实事、解难事，促进社会公平正义，维护社会和谐稳定，把发展成果更多体现在增进民生福祉上。

　　为完成2019年目标任务，重点做好以下工作：

　　一、深化市场化改革和开放合作，着力优化营商环境

　　以优化振兴发展营商环境为基础，加快市场化改革，扩大开放合作，努力打造优良的政务环境、市场环境、法治环境，努力做到“办事不求人”。

　　（一）深化政务服务改革。深入推进“互联网+政务服务”。以精减为目标，深化行政权力事项改革。继续精简权力事项。全面完成新一轮政府机构改革，深化事业单位分类改革。建立单一层级的重点领域行政综合执法体制。以智慧化为目标，深化服务方式改革。加快建设覆盖市县（区）乡（街道）三级的一体化在线政务服务平台，统一网络支撑、电子认证、数据共享和“一网通办、最多跑一次”标准流程。以便民为中心，扩大手机端、自助端服务应用，打造24小时网上受理服务平台。强化电子政务云信息安全监管服务。不动产登记最复杂事项办理不超过5个工作日、更多事项即时办结。丰富智慧场景应用，在卫生、教育、交通、养老、公益事业等领域积极探索，推动智能化智慧化社会服务。深化投资审批制度改革，推广园区项目建设承诺制零审批；深化“多规合一”“多审合一”，强化联办并行、中介服务的网上技术支撑，大幅度压缩投资审批时限。深化以事项为单元的商事制度改革，深入推进“多证合一”“联动联办”，探索实施承诺办、信用管，推行跨部门、跨层级的“一链通办”模式，扩大“一次办”覆盖面，企业开办时间压缩到8个工作日以内。对标中国营商环境指标体系，开展营商环境评价。以依法和信用双向监管为目标，深化行政管理方式改革。建设以数据为基础、以重点领域为主的在线监管平台。建设行政执法权力运行“数据铁笼”，探索推动跨部门联合执法，实施线上线下一体化综合监管，逐步实现数字监管事项全覆盖、监管过程全记录、监管数据可共享可分析可预警。深化社会信用体系建设，扩展市级信用信息平台应用，推进涉企信息资源共享，做实企业“红黑名单”制度，提升联合惩戒和联动激励治理效果。

　　（二）激发微观市场主体活力。以打破壁垒和激励引导为手段，建立健全优化营商环境的工作机制，促进各类市场主体竞相发展。深化国有企业市场化改革。以产权为核心，进退并举，积极稳妥推进市场化分类改革，解决历史遗留问题。组建市金融控股、城市建设、旅游集团，深化市粮食集团、城投公司、国投公司改革。优化国有资产布局，推进产权多元化，加快建立现代企业制度，完善国有资产监管体制。推动县（市）区属企业改革；支持服务中省属企业改革，全面完成驻齐央企“三供一业”分离移交。大力发展民营经济。降低市场准入门槛，落实国家扶持民营经济发展政策，出台我市支持民营经济发展实施意见，着力打破人才、金融等要素制约，营造公平竞争环境。建立民营经济发展协调服务机制，实施民营企业梯度成长培育计划，支持民营企业做优做大做强。深化要素市场化改革。落实市委各项人才政策，用好省就业创业扶持十二条政策，实施就业留人、创业聚才。加大贷款周转金扶持力度，加快普惠金融体系建设，推行政银企保合作，着力破解融资难融资贵问题。落实电力价格改革部署，降低企业用电成本。加强公共资源集中统一交易管理，处置盘活闲置土地和僵尸资产。统筹推进农村“三地一权”“三权分置”改革，在确权登记基础上，加快探索农业农村生产要素市场化流动，促进农业增效农民增收。深化供销社综合改革。加快“四上企业”培育。以释放潜量、盘活闲量、做优存量、引进增量为途径，以大众创业和个转企、小升规、规转股、股上市为重点，启动实施市场主体培育三年行动，促进生成更多的规模市场主体，新增规上工业企业25户、规模建筑施工企业40户、限上商贸企业30户、规上服务业企业25户，“四上企业”突破千户。新增资本市场挂牌企业10户以上。建立一批众创空间。

　　（三）加强开放合作。依托欧亚大陆桥重要节点，主动融入“一带一路”，加强与俄欧、东北亚的技术、资本和产品合作，提高经济外向度，外贸进出口总额增长10%。加快三间房国际经贸物流产业园区建设，建成进口肉类指定查验场，发挥跨境电商作用，发展代理出口、通关等居间服务，提高互联互通水平。培育壮大外向型企业，拓展建龙北钢、齐车、阜丰、景宏石油等企业进出口业务，推进一重、齐重数控、全联重型等企业国际化合作。坚持“走出去、引进来”，学习借鉴发达地区经验，加强企业间沟通合作，推动与广州对口园区和项目取得突破性进展，主动承接京津冀、长江经济带优质项目转移。深化开发区改革创新，实施百亿级产业园区攻坚，搭建产业项目聚集和开放合作共享平台。

　　二、培育壮大新动能，着力促进产业振兴

　　围绕做好“三篇大文章”，深入推进全市12个重点产业，实施“百万千”产业倍增计划，加快构建现代产业体系，打造新动能，促进新发展。

　　（一）聚焦高效生态农业打造新动能，加快现代农业振兴发展。以建设高效生态农业示范区为引领，推动绿色兴农、效益兴农，担起国家粮食安全重任，争当全省农业现代化建设排头兵。加快培育新型农业市场主体。巩固发展种养大户、家庭农场，新增一体化合作社20个、股份合作组织50个、联合社26个、农业公司20个。引进建设战略性农业龙头企业，促进农业生产经营规模化、市场化、产业化。推动益海嘉里、阜丰、鸿展、中粮等重点项目建设和企业创新发展。按照“稳生猪、优两牛”的思路，推进“两牛一猪”标准化规模养殖，推动飞鹤、元盛、嘉一香、东方希望等龙头企业创新发展，加快标准化规模养殖和加工项目建设，完善种养加销全产业链，打造国家高质量乳肉产业基地。加快农业科技创新。强化农业科技创新体系和社会化服务体系建设，坚持市场导向调结构、优品种、提品质，增加蔬菜、食用菌、中草药等优质高效经济作物。加快国家级大豆育种基地、寒地粳稻育种基地建设，推广百项农作物优良品种和新技术，升级现代农业科技产业园35个。巩固千万亩绿色有机食品基地，新增生态高标准农田55万亩。新增高效节水灌溉面积30万亩以上，提升有效灌溉能力。完善畜禽良种繁育推广和防疫保障体系，加快“15吨高产奶牛”“龙江和牛”“天兆种猪”等优良品种扩繁，推进规模化迈向高效化优质化。打好防控非洲猪瘟疫情持久战。推动一二三产融合。发挥原生态优势，新生成一批绿色有机、寒地黑土、非转基因地域品牌，厚植三次产业融合；突出“两头两尾”产业项目建设，提高农产品加工转化率，带动三次产业融合；深入推进“互联网+农业”，大力发展农产品电商和区域营销合作，拓展域外市场，促进三次产业融合。深化“农村客运+电商物流+交邮合作”等多种模式，解决农村物流短板。培育发展一批宜居宜业宜游、产城融合的特色小镇、休闲农业、生态田园综合体等新业态。

　　（二）聚焦智能化服务化打造新动能，加快先进制造业振兴发展。坚持“五化”创新发展方向，以制造业智能化、服务化为引领，带动高端化、绿色化、国际化发展，加快打造装备、食品两大千亿级产业集群，增强工业核心竞争力，加快建设工业强市。加快高质量产业项目招商建设创新。启动新一轮百个重点产业项目三年攻坚，聚焦12个重点产业，突出龙头打造、产业配套和园区集约发展，坚持新建与改造并举，大上有就业、有税收、有市场、有产业带动的高质量项目，推行“领导+项目”“专班+园区”“目标+考核”的招商引资新机制，抓实项目开工率、投资率、达产达效率。推进一重和建龙北钢升级改造、紫金铜产业园、腾翔铸锻造等重点项目建设。加快工业科技创新。协同推进哈大齐国家自主创新示范区建设，启动新一轮科技型企业三年攻坚。以产业需求为导向，加快产学研融合集聚，新建一批国家和省科创平台、孵化加速器。培育壮大创新型产业联盟，强化运行实效。实施重大关键核心技术攻坚。实施“头雁行动”，建设国家级高端人才双创平台，做实“筑巢引凤”，吸引更多高端人才和领军团队为我所用。强化高技能人才培养，打造工匠摇篮。推动科技成果生成、引进和产业化，落地转化科技成果345项，培育成长型科技中小企业200户，新增国家级高新企业10户、科技型企业90户、创新型领军企业2户。高新智谷创建国家级孵化器，入孵项目达到120个，做实“一区三园”，提升高新区创新发展的集聚带动能力。推动制造业智能化服务化融合发展。加快制造业智能化改造，拓展设备租赁、远程运维、工艺升级和供给服务新业态，通过智能化促进服务化，推动先进制造业和现代服务业深度融合。新增数字化车间10个，推动一批企业上云。启动国家装备制造业智能化服务化三年行动示范试点。加大军工科研技术溢出，推动军转民、民参军、军民融合深度发展。

　　（三）聚焦新业态新模式打造新动能，加快服务业振兴发展。坚持电商化、便捷化发展方向，推动服务业业态模式创新，着力打造区域商贸物流、旅游、文化和医疗等中心，培育壮大千亿级服务产业集群，拓展千万人消费市场，加快建设东北地区西部区域中心城市。加快商贸物流业发展。推动电商与实体商贸物流业深度融合，选树一批现代商贸物流示范企业，带动传统重点商贸企业业态模式改造升级。推进一批综合新业态项目建设，满足引领消费需求，增强域内供给力，拓展域外辐射度。引导商贸物流企业应用大数据平台，融入全国大市场发展体系。新增电商企业500户，上行交易额增长20%；限上企业触网率达到80%。加快旅游、文化、体育、生态、养老产业融合发展。围绕建设旅游强市目标，以发展全域旅游、四季旅游为重点，优化旅游产业布局，巩固“4+1”城际旅游联盟，突出鹤品牌，打造全省西线旅游龙头。挖掘旅游资源，开发更多生态产品，推动文化精品商演，叫响冰球城市品牌，办好冰球节、冰球季和各类赛事，打造冰雪运动和装备基地，发展多元化养老服务，吸聚更多域外旅游消费。坚持旅游资源景区化、景区运营市场化，推动扎龙湿地等旅游景区做大做强。巩固团组游、自助自驾游，大力发展研学游、亲子游。推进旅游服务电商化，做实线上线下一体化服务。加快综合业态景区、旅游配套服务项目建设。全年接待游客和旅游总收入分别增长10%以上。加快新型生产性服务业发展。加快“大智移云”新一代信息技术应用，创新发展生产性服务业。引进人工智能、互联网、区块链等战略性技术服务项目落地，支持浪潮、九次方等互联网企业在我市做大做强，加大对信息、技术、中介、金融等传统生产性服务业智能化市场化升级改造，培育发展数字经济、共享经济，以智能技术专业服务引领产业创新。

　　（四）突出“两翼”协调发展打造新动能，加快全域经济高质量发展。全面落实《城区经济发展指导意见》，大力发展先进制造业和现代服务业，发挥城区经济引领作用，带动全域经济高质量发展。突出解决县域经济短板，深入实施乡村振兴战略，围绕农产品资源优势、产业基础优势和土地、人力资源等要素优势，大力发展食品、农副产品精深加工项目，做大二产、带动一产、助推三产，优化县域经济结构，扩大规模，提升速度，发挥县域经济基础作用，支撑全域经济高质量发展。

　　三、强化生态文明建设，着力保障生态安全

　　深入开展污染防治攻坚三年行动，强化生态环境治理保护，建设美丽鹤城、生态强市。

　　（一）打赢蓝天保卫战。深入实施燃煤污染专项治理，加快淘汰城镇10蒸吨及以下燃煤锅炉，原则上不再新建35蒸吨以下燃煤锅炉，城区、县城集中供热普及率分别达到95%和90%以上；深入实施秸秆污染专项治理，继续实行全面禁烧，提高秸秆离田率和综合利用率；深入实施扬尘面源污染专项治理，加强施工现场和堆场粉状物料堆放、作业、运输全过程管控；深入实施移动污染源专项治理，淘汰高污染排放车辆580台；深入实施工业污染源专项治理，清理整顿“散乱污”企业，探索排污许可管理；深入实施供暖季重污染天气专项治理，强化用煤企业管控，提升应急响应处理能力。全年空气质量优良天数保持在90%以上。

　　（二）打好碧水保卫战。深入实施饮用水保护专项治理，饮用水水源地全面整治达标；深入实施城市生活污水专项治理，城镇污水处理厂全部达到一级A稳定排放标准，提高污水收集处理能力，完成重点黑臭水体整治；深入实施工业污水达标专项治理，推进涉水工业企业主要污染物减排；深入实施畜禽粪污专项治理，规模养殖场粪污处理设施配套率、设施运行率和资源化综合利用率分别达到95%、100%和75%；深入实施流域水污染防治专项治理，全面建立河湖长制“一河一策、一河一规划、一河一巡护、一河一监测”长效体系，深入开展以“四清”为重点的清河行动，继续推进乌裕尔河、双阳河流域生态补偿，建立嫩江流域生态补偿机制，确保六个国控考核断面水质全部达标。

　　（三）推进净土保卫战。深入实施黑土地保护专项治理，继续开展轮作休耕、免耕和测土配方施肥，持续推进国土绿化行动和三北防护林工程，实施退耕还林还草还湿，植树造林5.5万亩，完成昊华电石渣老堆场土壤污染治理修复；深入实施农业土壤污染专项治理，农业“三减”5%以上，废旧农膜清理回收率达到78%；深入实施危险固体废物专项治理，推进工业企业标准化固废堆场建设，加快红星等老旧堆场搬迁治理，生活垃圾无害化处理率达到85%以上，污泥处置率达到100%；深入实施“三线一单”空间管控专项治理，完成生态环境总体规划编制，建立环境准入负面清单管控体系。

　　（四）实施区域环境综合治理。推进自然保护区综合治理。以问题点位排查整改和生态修复为重点，持续深化“绿盾行动”，加强一般湿地治理，推进扎龙湿地、乌裕尔河湿地治理修复。倡导绿色生产生活。培育发展节能环保产业，推进生态、循环和碳汇经济发展，建设国家可再生能源综合应用示范区。倡导绿色低碳生活方式，深化绿色家庭、绿色校园、绿色社区、绿色出行等创建行动。

　　（五）强化生态环境保护。坚决守住生态保护红线，统筹山水林田湖草综合保护，积极推进自然资源用途管控，严禁新建环保不达标项目，严控高耗能高污染项目审批。谋划推进松辽平原西南季风通道风沙综合治理等项目。强化“天地一体化”管控，利用互联网和遥感卫片技术，加快生态环境大数据监管平台建设，对重点区域、重点污染源实行数据和视频在线监管；建立健全保护区和流域专兼职管护队伍，整合下沉生态环境保护综合执法队伍。推动自然资源资产管理和有偿使用制度改革，强化自然资源资产责任审计，严格落实生态环境损害责任追究制度，严厉打击生态环境违法犯罪。完成第三次国土调查。

　　四、持续推进“七大民生工程”，着力办好民生实事

　　持续推进“七大民生工程”，一年接着一年干，一件接着一件办，不断增强群众获得感幸福感安全感。

（一）持续推进脱贫攻坚工程。把提高脱贫质量放在首位，聚焦“两不愁、三保障”突出问题，巩固提升脱贫成果。做实产业扶贫，培育新型经营主体，壮大集体经济，搞活“小院经济”，优化扶贫产业项目，提升产业持续带贫能力。注重实效，彻底解决医疗保障、住房保障和饮水安全“三大短板”，统筹建立扶贫综合保障长效机制。强化扶贫资金全程监管审计，提高扶贫资金使用绩效。完成龙江、克东、泰来3个县脱贫验收，集中力量支持拜泉深度贫困县达到脱贫退出条件，确保26个贫困村全部出列，2.53万贫困人口全部稳定脱贫，力争提前一年完成脱贫攻坚任务。

（二）持续推进社会保障工程。深入开展就业服务行动。强化农村剩余劳动力培训引导和校企共建，畅通就业供需通道，做实订单培养和双元培育，构建与产业发展相协调的人力资源开发体系，大力推动促进型就业，新增城镇就业8万人以上。落实高校毕业生就业创业实施意见，扶持7000名大学生就业创业。强化援企稳岗政策效应。建立保障农民工工资支付长效机制。深入开展扩面提待行动。扩大养老保险覆盖面，按政策提高并及时足额发放养老金。提高城乡居民医保标准，大病医疗费用报销比例上调3%，城乡居民门诊慢性病增加到14种；扩大特殊药品使用目录，国家谈判特殊药品纳入门诊报销范围。深入开展社会救助行动。按政策提高城乡低保和特困人员救助标准。落实残疾人辅助器具适配项目，强化残疾人基本康复服务。做好救灾减灾工作，争创国家、省级综合减灾示范社区7个。

　　（三）持续推进宜居工程。深入开展保障房改造行动。全市棚改回迁安置2万户以上，改造农村危房4000户。深入开展老旧小区综合改造行动。改造“三水”管网50公里、低温楼6万平方米。深入开展保供行动。启动富拉尔基、铁路新水源和城区管网改造项目建设，强化二次加压泵站管理。扩大城区热电联产集中供热覆盖率，积极推进清洁供暖。提高燃气管网覆盖率。深入开展物业服务提升行动。出台物业服务等级标准，规范物业服务市场经营秩序，提供质价相符的优质服务。建立健全“物业+业主+社区”联动机制，推行双向信用惩戒机制，构建和谐的物业供需关系。深入开展住房为民行动。加快解决房地产开发历史遗留问题。科学供应土地，增加优质商品房供给，促进房地产市场供需平衡。

　　（四）持续推进城市交通改善工程。深入开展路网优化行动。打通一批道路微循环，推进道路渠化改造，完成中心城区腾飞路外环路建设，开工建设富拉尔基富江路跨线桥。深入开展停车规范行动。新增和开放一批停车位，破解校园周边停车难题，鼓励建设复式停车设施，强化管理运营，提高停车设施周转率。深入开展公交升级行动。推进公交专用道和充电首末站建设，优化公交站点和线路，提升公交服务质量，提高公交分担率。深入开展管理提效行动。利用大数据，科学完善交通组织，优化交通管理设施，强化执法监管，提高道路畅通率。续建改造2条国省道，全面启动机场改扩建。

　　（五）持续推进城市环境整治工程。推进城市环境整治向小区、城中村、近郊村延伸，实现城区环境由干净整洁向生态优美转变。深入开展全民绿化行动。绿化提升城区道路42条、小区庭院24个、公园绿地7处。深入开展全城亮化行动。突出建管并重，建立亮化管护责任机制。抓好公建单位、新建小区、广场公园等重点区域亮化美化。深入开展清洁行动。继续开展“四季环境整治”。突出精细化管理，强化清洁网格责任制，完善环卫基础设施，提高湿扫率。深入开展市容整治行动。突出严管严治，以清理“八乱”为重点，有效整治店外经营、路面造脏、物料堆放等乱象。规范管理烟花燃放、祭祀烧纸。启动垃圾集中分类处理行动。编制城乡垃圾综合治理专项规划，全面推行餐厨、建筑、医疗垃圾分类处理，探索生活垃圾分类收集，加快垃圾焚烧发电项目建设。启动拆违整治行动。坚决清理存量、遏制增量，城区拆除违建1.2万平方米以上。按时完成“大棚房”整治。

　　（六）持续推进社会事业提升工程。深入开展教育为民行动。以办好人民满意教育为宗旨，强化师德师风建设，稳步推进中高考改革，提高课堂教学质量，全面推进满足需求的课后服务，整治校外补课乱象，着力解决“大班额”等问题，继续推行“阳光分班”。巩固义务教育基本均衡县建设成果，统筹发展学前教育，办好公平而有质量的教育。深入开展健康鹤城行动。以解决看病难看病贵为着力点，深化公立医院改革，推动医联体、医共体建设，健全分级诊疗机制，注重培养全科医生，做实家庭医生签约服务，强化基层卫生保障能力。提高中医药服务能力。第一医院南院建成投用。规范药品采购，推行总额预付、单病种结算管理方式，提升医保基金使用效率。普及健康知识，倡导健康生活方式，广泛开展全民健身，新增健身器材200套以上。深入开展文化惠民行动。完善城乡公共文化服务体系。办好庆祝中华人民共和国成立70周年系列文化活动，开展“鹤城百姓文化季”“龙沙音乐会”等百场群众文化活动，文艺院团常态化演出230场。推进卜奎清真寺、清末藏书楼等重点文物保护利用。

　　（七）持续推进农村人居环境整治工程。以建立健全农村公共服务体系为抓手、以整村推进为重点，着力推进农村垃圾污水处理、厕所革命、能源革命和村容村貌整体提升。基本建成农村垃圾收运处体系。多渠道建立污水粪污处理长效机制，改厕1.3万个。加大改灶力度，推广清洁燃料。绿化行政村279个，新建农村公路400公里，解决130个村屯饮水安全问题，改造一批村内设施，建立完善村屯环境治理长效机制。加快农村人居环境达标建设，中心村基本实现达标，中心屯、基础较差的行政村规范整治率40%以上。

　　五、加强政府自身建设，着力提升现代治理能力

　　面对全面振兴全方位振兴高质量发展的新要求新期待，必须坚持党的全面领导，树牢“四个意识”，坚定“四个自信”，做到“两个维护”，着力增强政府执行力、服务力、公信力，以新气象新担当新作为，承担起政治责任和时代责任。强化思想解放。深入开展解放思想推动高质量发展大讨论，以思想大解放促进观念大更新、能力大提升、工作大创新，强化互联网思维、改革创新精神、市场意识和专业水准，增强系统化解决短板弱项的能力。强化法治政府建设。严格遵守宪法法律，深化学法制度，确保政府工作始终在法治轨道上运行。依法接受人大法律监督和工作监督，自觉接受政协民主监督，主动接受社会监督和舆论监督，认真办理人大代表议案、建议和政协委员提案，使政府工作更好地体现群众意愿。强化社会治理创新。加快构建立体化信息化治安防控体系，强力推进“扫黑除恶”专项斗争，强化信访维稳和社会矛盾化解攻坚，加强应急救援体系建设，严格落实安全生产责任制，坚决防范重特大事故。加强食品药品监管，保障群众饮食用药安全。抓好双拥共建，创新社会组织管理，推进城乡社区标准化建设。强化廉政勤政。严格落实政府系统党风廉政建设主体责任和“一岗双责”，认真贯彻中央八项规定及实施细则精神，持续深化作风整顿优化营商环境活动，坚决克服和防止形式主义、官僚主义。健全督查激励问责机制，支持褒奖干部敢担当真作为，严厉惩戒庸政懒政怠政行为。强化巡视巡察和审计结果运用，加强财政资金、政府项目等重点领域管控，推进政府信息公开，促进廉洁用权。有效防范化解地方政府债务风险。牢固树立过紧日子思想，强化增收节支，建立税源建设推进机制，严控“三公”经费，把有限财力用在保基本、惠民生、促振兴上。

　　各位代表！让我们高举习近平新时代中国特色社会主义思想伟大旗帜，更加紧密地团结在以习近平同志为核心的党中央周围，在省委、省政府和市委的坚强领导下，解放思想，改革创新，锐意进取，担当作为，为鹤城全面振兴全方位振兴高质量发展而不懈奋斗，以优异成绩迎接新中国成立70周年！
